# A Model for Cell Wall Dissolution in Mating Yeast Cells: Polarized Secretion and Restricted Diffusion of Cell Wall Remodeling Enzymes Induces Local Dissolution

**DOI:** 10.1371/journal.pone.0109780

**Published:** 2014-10-16

**Authors:** Lori B. Huberman, Andrew W. Murray

**Affiliations:** 1 Molecular and Cellular Biology, Harvard University, Cambridge, MA, United States of America; 2 Faculty of Arts and Sciences Center for Systems Biology, Harvard University, Cambridge, MA, United States of America; Florida State University, United States of America

## Abstract

Mating of the budding yeast, *Saccharomyces cerevisiae*, occurs when two haploid cells of opposite mating types signal using reciprocal pheromones and receptors, grow towards each other, and fuse to form a single diploid cell. To fuse, both cells dissolve their cell walls at the point of contact. This event must be carefully controlled because the osmotic pressure differential between the cytoplasm and extracellular environment causes cells with unprotected plasma membranes to lyse. If the cell wall-degrading enzymes diffuse through the cell wall, their concentration would rise when two cells touched each other, such as when two pheromone-stimulated cells adhere to each other via mating agglutinins. At the surfaces that touch, the enzymes must diffuse laterally through the wall before they can escape into the medium, increasing the time the enzymes spend in the cell wall, and thus raising their concentration at the point of attachment and restricting cell wall dissolution to points where cells touch each other. We tested this hypothesis by studying pheromone treated cells confined between two solid, impermeable surfaces. This confinement increases the frequency of pheromone-induced cell death, and this effect is diminished by reducing the osmotic pressure difference across the cell wall or by deleting putative cell wall glucanases and other genes necessary for efficient cell wall fusion. Our results support the model that pheromone-induced cell death is the result of a contact-driven increase in the local concentration of cell wall remodeling enzymes and suggest that this process plays an important role in regulating cell wall dissolution and fusion in mating cells.

## Introduction

Cell fusion is essential for sexual reproduction and plays an important role in the development of many organisms [1]. In mammals, cell fusion is involved in the formation of myoblasts [2], osteoclasts [3], giant cells [4], and placental cells [5]. It is also important in the development of *Caenorhabditis elegans* [6] and *Drosophila melanogaster* [7]. Perhaps the simplest and most well studied form of cell fusion is the mating of the budding yeast, *Saccharomyces cerevisiae* [8].

Budding yeast can exist in both a diploid and haploid state. In either state, cells can replicate asexually by budding, producing daughters that are genetically identical to their mothers [9]. Haploid cells can be one of two mating types, **a** or α, which are defined by two alternative alleles of a single locus, *MAT* **a** or *MAT*α. These mating types express reciprocal pheromones and pheromone receptors, which they use to signal to each other. Exposing a *MAT* **a** cell to α-factor, the pheromone secreted by *MAT*α cells, (or vice versa) induces a pheromone response that includes transcription of pheromone response genes, cell cycle arrest in G1, and polarization in the direction of highest pheromone concentration to form a mating projection known as a shmoo [10].

After *MAT* **a** and *MAT*α cells have successfully communicated and grown towards each other, they must fuse [9]. The two cells initially bind to each other at their shmoo tips using mating agglutinins [11]–[13], but their plasma membranes are still separated by two, approximately 100nm thick, cell walls [14]. Before the mating partners can fuse, the cell wall that lies between the two membranes must be dissolved and the boundaries of the remaining cell walls, which surround the site of cell fusion, must fuse to form a single, continuous structure that will enclose the newly formed zygote [8]. The osmotic pressure differential between the cytoplasm and the extracellular environment makes this spatially regulated cell wall dissolution and fusion a dangerous task [15], [16]. If the cell wall is opened at the wrong time or place, exposing the plasma membrane directly to the environment, there will be no elastic force to resist the turgor pressure of the cell, water will rush into the cell from the extracellular environment, and the cell will lyse [15], [16].

Various studies have been done on the molecular basis for cell wall dissolution. In 1996, Brizzio *et al.* showed that high pheromone concentrations are required for efficient fusion and hypothesized that vesicles found at the shmoo tip might contain cell wall remodeling enzymes [17]. Later, Cappellaro *et al.* found several proteins with homology to known cell wall glucanases, including *SCW4*, whose deletion makes mating less efficient [18]. The promoter of another putative glucanase gene identified by Cappellaro *et al.* [18], *SCW11*, has a binding site for Ste12 [19], the transcription factor that induces genes in response to pheromone stimulation [20].

Several proteins that are required for efficient cell fusion play a role in delivering secretory vesicles to the shmoo tip [21]–[24]. A complex containing Rvs161, an amphiphysin-like protein that binds curved membranes [25], [26], and Fus2 [22], [27], [28], is hypothesized to direct vesicle transport to the cell fusion zone [23]. Once the vesicles reach the plasma membrane, they are anchored by Fus1 [23], a membrane spanning protein [29] that interacts with the polarisome [30], a protein complex associated with polarized actin polymerization [31], presumably ensuring tight clustering of the secretory vesicles. Although these proteins direct vesicle secretion towards the shmoo tip, their roles do not explain how cell wall dissolution is limited to the site of contact with a polarized partner.

**Figure 1:**
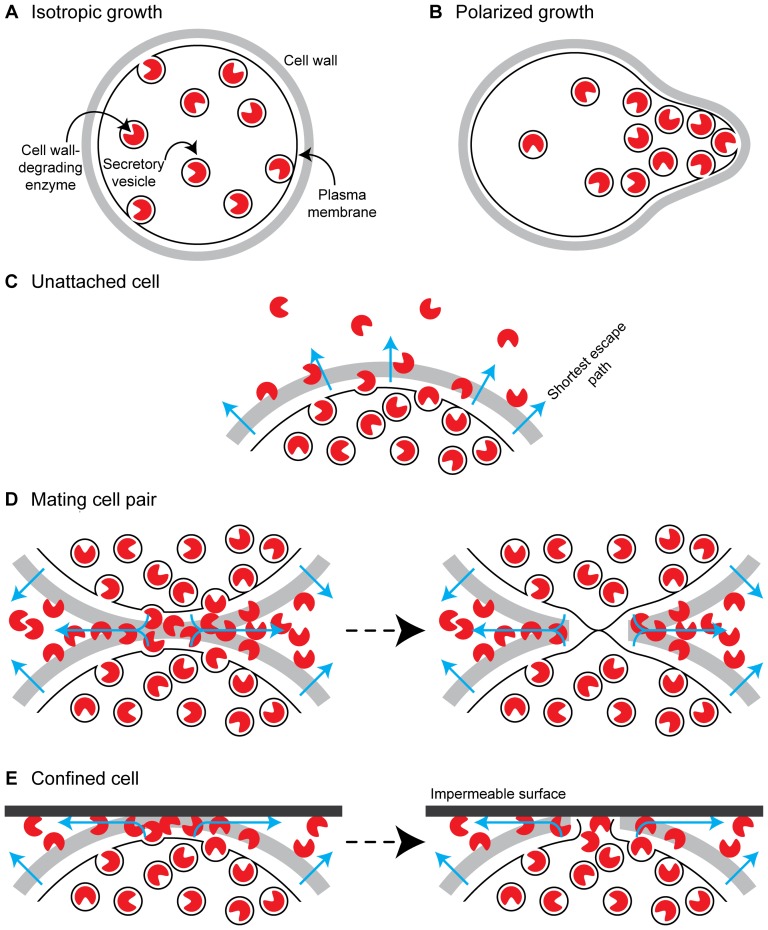
Model: Confining cell wall degrading enzymes in the fusion zone leads to cell wall destruction. **A.** Isotropically growing cells increase the size of their cell walls equally in all directions to grow larger while maintaining an ellipsoidal shape, so cell wall remodeling enzymes are secreted equally in all directions. **B.** Polarized cells grow anisotropically, so they polarize secretion of cell wall remodeling enzymes to expand their cell walls in the direction of polarization. **C.** When pheromone stimulated cells are unattached, the cell wall remodeling enzymes secreted from the shmoo tip exit the cell wall along the shortest path by traveling perpendicular to the plasma membrane. These enzymes break cell wall bonds as they diffuse through the wall to allow continual expansion of the shmoo up the pheromone gradient, but the wall is not breached. **D.** When two pheromone-stimulated cells are attached by mating agglutinins, the cell wall remodeling enzymes secreted into the future fusion zone must now travel further to exit the cell wall, traveling parallel to the plasma membrane until they reach the edge of the agglutinated zone, increasing the local concentration of cell wall remodeling enzymes in this zone. The cell wall remodeling enzymes dissolve the two cell walls at the point of contact while cell wall synthesizing enzymes simultaneously interlock them, allowing the plasma membranes of the two cells to contact one another and fuse without exposing the cell to osmotic lysis. **E.** We mimicked the attachment of two cells by tightly apposing single cells to impermeable surfaces, forcing cell wall remodeling enzymes to exit the wall by traveling parallel to the plasma membrane until they reached bulk solution and thus increasing the concentration of cell wall remodeling enzymes at the point of attachment to the impermeable surface. This causes a hole to form in the cell wall, exposing the plasma membrane to the extracellular environment and causing the cell to undergo osmotic lysis.

The problem of remodeling the cell wall is not unique to mating. Even when cells are growing isotropically, there must be a balance between cell wall synthesis and destruction to allow the continual increase in cell diameter and volume, which is accomplished through spatially uniform secretion of synthesizing and remodeling enzymes (Figure 1A) [32], [33]. Polarized growth, such as that associated with budding and shmooing, is achieved through polarized secretion of these enzymes [33] (Figure 1B). Most cell wall synthesizing enzymes are attached to the plasma membrane, whereas most wall-degrading enzymes are free to diffuse through the cell wall [34]. Synthesis and destruction must be carefully balanced: an excess of synthesis over degradation will lead to an increased cell wall thickness and eventually to slow growth, whereas an excess of degradation will weaken the cell wall until it is unable to resist the osmotic pressure inside the cell [35].

We propose a simple model to explain how cell walls are dissolved at the point where two polarized mating partners contact each other. When cells are not stuck to each other by mating agglutinins, the degradative enzymes diffuse through the cell wall and are then lost into the medium (Figure 1C). But when two mating partners stick to each other, using agglutinins, the enzymes must take a much longer path to escape, and because distance diffused only rises as the square root of time, their concentration at the site of fusion must rise, leading to an excess of destruction over synthesis and the eventual dissolution of the cell wall (Figure 1D).

If our model is correct, it should be possible to cause pheromone-induced cell death by tightly apposing pheromone-treated cells to impermeable surfaces, thus, mimicking the attachment of two cells to each other during mating (Figure 1E). Although previous studies [36], [37] have reported that pheromone treatment can cause cell death, they neither hypothesized a mechanism through which this process is regulated, nor carefully examined the effect of holding cells against impermeable surfaces. We therefore set out to test the idea that slowing the escape of cell wall-degrading enzymes would lead to cell wall dissolution and death.

We observed that the frequency of cell death increases as the amount of cell contact with an impermeable surface increases and as the osmotic pressure differential between a cell and its environment rises, whereas decreasing the osmotic pressure differential reduces cell death. Deleting Fus1 and Fus2, proteins important for cell wall fusion [24], as well as the putative cell wall glucanases Scw4 and Scw11 [18], also decreases the frequency of cell death. Our evidence argues that the pheromone-induced cell death is due to a contact-dependent increase in the local concentration of cell wall remodeling enzymes, leading to the dissolution of the cell wall and eventual lysis of the cell. This mechanism may ensure safe and accurate cell wall fusion during mating.

## Results

### A model for pheromone-stimulated cell wall dissolution

We propose a simple model for cell wall dissolution: cell-cell contact increases the concentration of cell wall remodeling enzymes because they have to diffuse further within the cell wall from their site of secretion to reach the aqueous solution that surrounds the cells. We mathematically analyzed the distribution of cell wall remodeling enzymes in two situations: cells that are free in aqueous medium and those apposed to an impermeable surface. In both cases, we assume that the enzymes diffuse much more slowly through the cell wall than they do in the surrounding medium, and that this medium represents an infinite sink, allowing us to set the enzyme concentration outside the cell wall to zero. For unapposed cells, the enzymes need only diffuse through the thickness of the cell wall. At steady state, the flux through all points from the external surface of the plasma membrane to the external surface of the cell wall must be constant, implying a linear gradient in the enzymes' concentration. For apposed cells, we assume that they have a circular area of the cell wall pressed against an impermeable surface and that secreted enzymes must diffuse through the wall, parallel to the impermeable surface, before they can escape. At the center of the apposed region, the cell is secreting enzymes into the wall and the enzymes are diffusing away from the center of this region. In this region, the flux through the circumference of circles inscribed in the cell wall increases as the radius of the circles increases. Because the area of secretory activity increases with the square of the radius, whereas its circumference increases only linearly, the flux per unit length of the circumference increases, and thus the steepness of the gradient increases, moving outwards from the center of the secretory zone. Beyond this zone, no new enzyme secretion occurs, the flux through successively larger circles remains constant, and since their circumference increases, the radial concentration gradient becomes progressively shallower. If we assume that the cell wall is 0.1 µm thick, the radius of the secretory zone is 0.25 µm, and the radius of the apposed zone is 1 µm, the concentration of cell wall degrading enzymes at the center of the apposed zone is more than ten times the mean enzyme concentration in the wall of an unapposed cell. Details of this analysis are found below.

### Mathematical analysis of pheromone-stimulated cell wall dissolution

First we consider an enzyme diffusing one dimensionally through a cell wall with diffusion coefficient *D*. If the radius of the secretion zone is substantially greater than the thickness of the cell wall, it is reasonable to treat the escape of enzymes secreted at the center of this zone as proceeding by one-dimensional diffusion through the thickness of the cell wall. As it diffuses through the wall, the enzyme's flux through a unit area of the cell wall, parallel to the surface of the cell, *J*, must be constant and is given by Fick's law
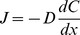
where *x* is the distance along the axis that runs perpendicular to the external surface of the plasma membrane to the outer surface of the cell wall. Since the flux is constant at all the points along this axis, the gradient must be the same at all points through the thickness of the wall, and thus the total concentration difference across the wall *ΔC* must increase linearly with the thickness of the wall, *ΔC* = *x/D*. If we set *D* within the cell wall to be much lower than it is in solution, the concentration outside the wall will be close to zero, and *C_0_*, the concentration at the site of secretion will be given by *C_0_* = *Jx/D*, and if we set the units of *x* to the thickness of the cell wall (roughly 100 nm), *C_0_* = *J/D*.

Now we consider an apposed cell, in which diffusion proceeds radially, in the plane of the cell wall, from the site of secretion to the edge of the apposed area. We consider two concentric regions within the opposed area, a central one where both secretion and diffusion occurs, and a peripheral one, where there is just diffusion. Remembering that we have set the unit of length equal to the thickness of the cell wall, within the region with secretion and diffusion, the flux that must leave an area of radius *r* is
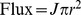
where *J* is the rate of enzyme secretion per unit area. At any radius, *r*, within the secretory zone, the enzyme must pass through a ring whose area is 2*πr*, specifying the radial concentration gradient, according to Fick's Law, and then, by integration, the concentration difference between the center (*C_0_*) and any radial distance, *r*, within the secretion zone,
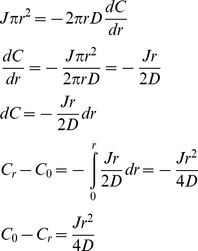
To get the concentration difference between the center and edge of this region, which is at *r_s_*, the radius of the secreting region, we substitute *r_s_* for *r*.
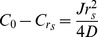



In the region where there is just diffusion, we can calculate the concentration difference from the inside edge to the outside edge of the region. Within the region, we must satisfy the condition that the total flux through each successive circumference is equal to the rate of enzyme production over the total producing region, which is 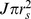. Thus for all *r*>*r_s_*,
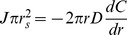
We can rearrange and integrate to get the change in concentration between the concentration at *r_s_* and *r*.
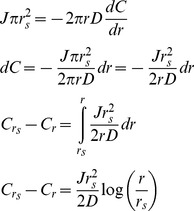



The overall concentration drop from the center of the secreting region to the edge of the apposed area is then given by adding the concentration drop from the center to the edge of the secreting region and the drop from the edge of the secreting region to the edge of the apposed region, situated at *r_max_*.
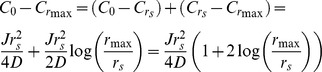
If we set *r_s_* = 2.5, and *r_max_* = 10, corresponding to radii of secretion and apposition of 0.25 and 1 µm, and consider the concentration drop from the center of the secreted region to its edge, *C_s_*, we get
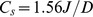
and for the drop from the outer edge of the secreted region to the edge of the apposed region, *C_d_*, we get
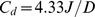
giving a total concentration drop, *C_tot_* = 5.89*J/D*, which implies a maximum hydrolase concentration that is nearly six times that attained when a cell is not apposed to another cell.

**Figure 2:**
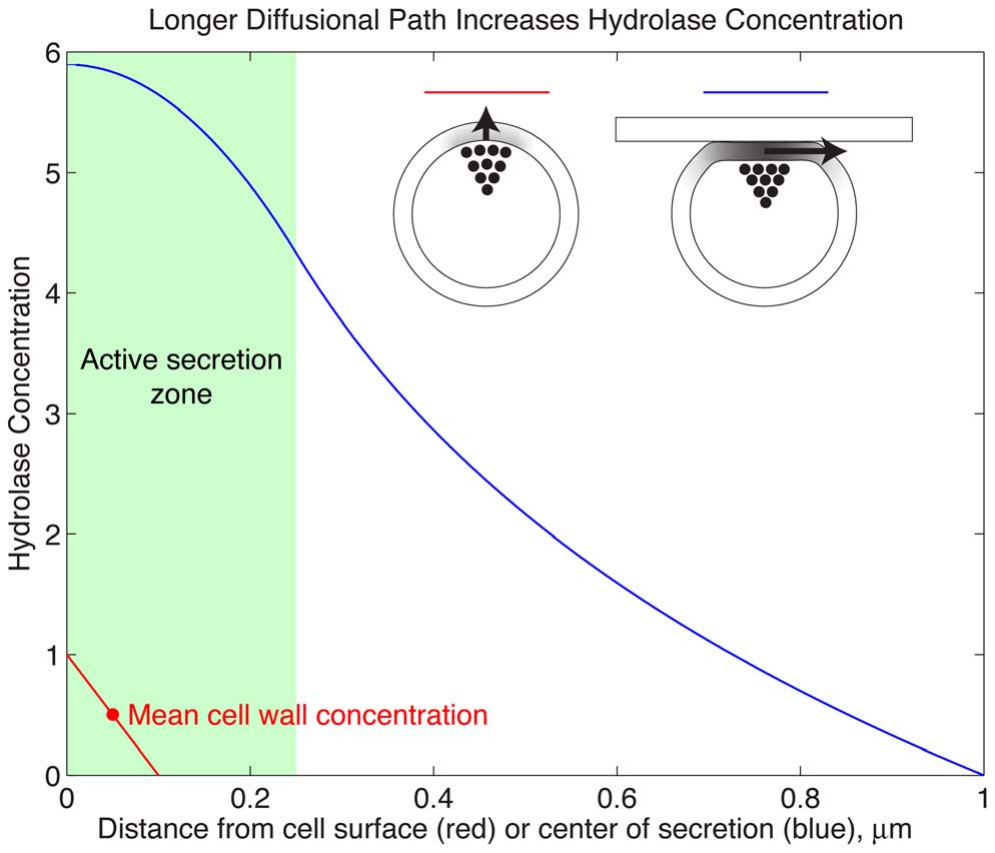
The role of radial diffusion through the cell wall of apposed cells in increasing the concentration of cell wall degrading enzymes. The graph shows analytical results for the relative enzyme concentration in two scenarios: *red*, diffusion through the cell wall, perpendicular to the cell surface, of a cell free in solution and not in contact with other cells or solid surfaces, and *blue*, diffusion through the cell wall, parallel to the cell surface, of a cell that is apposed to a solid surface, with a circular contact area whose radius is 1 µm.

The difference between the mean concentration at the center of the apposed region and the mean concentration in the wall of an unapposed cell is even higher. In unapposed cells, the mean concentration, felt half way through the cell wall, is *J/2D*, which is the average of a concentration *J/D* at the cell surface and 0 at the interface between the wall and solution. In the apposed cell however, the surface that the cell is exposed to acts as a reflecting barrier so that the concentration is constant across the thickness of the wall, and thus the mean concentration, in the scenario we have described is more than ten times higher for the apposed than for the unapposed cells (Figure 2).

### Pheromone-stimulated cells die when attached to an impermeable surface

**Figure 3:**
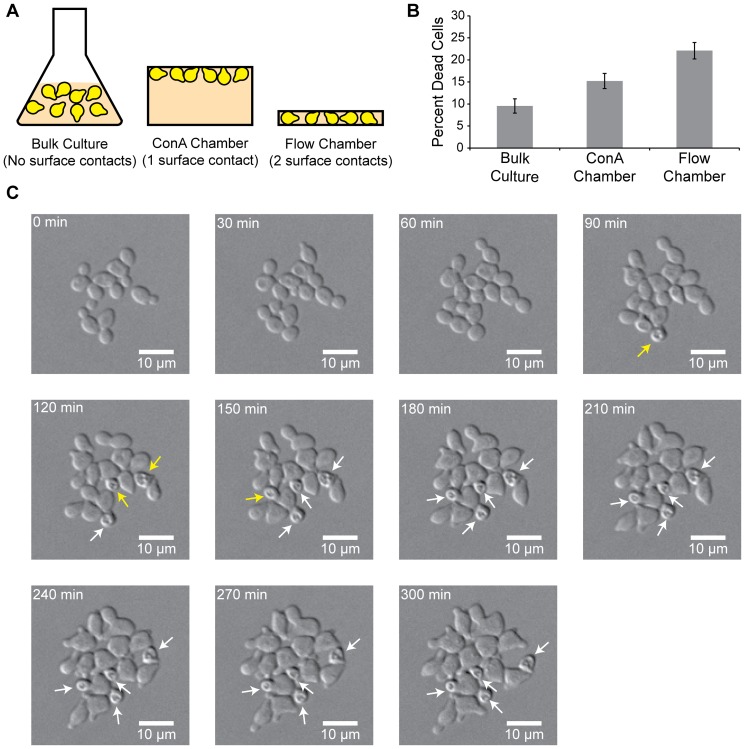
Pheromone-induced cell death increases with increasing attachments to an impermeable surface. **A.** Cells grown in bulk culture were incubated in test tubes on roller drums in liquid media without any enforced contact with impermeable surfaces. Cells grown in a concanavalin A (ConA) chamber were grown in a chamber whose depth was many times the diameter of a single yeast cell and attached to a single surface of the chamber (the ceiling provided by a glass coverslip) using the lectin, concanavalin A. For confinement, cells were loaded into a microfluidic chamber which traps cells between a ceiling and floor separated by the diameter of a single yeast cell, causing enforced contact with two surfaces. Medium is then constantly perfused through the chamber. **B.** Percent of *MAT* **a** *bar1Δ* cells that died after exposure to 50nM α-factor for five hours in three different physical environments. Error bars represent the standard deviation of at least three independent experiments. **C.** Time course of *MAT* **a** *bar1Δ* cells incubated in 50nM α-factor for the indicated amount of time in the flow chamber. Yellow arrows indicate cells that died since the previous time point. White arrows indicate cells that died earlier. The scale bar indicates 10 µm.

If contact with another cell leads to cell wall dissolution by increasing the local concentration of wall-degrading enzymes, we should be able to mimic the phenomenon by confining cells against an impermeable surface. We compared the response of pheromone-stimulated cells in environments where the cells were either free-floating, simulating cells in a mating mixture that are not attached to a fusion partner, or attached to an impermeable surface, simulating cells attached to a fusion partner via mating agglutinins. We observed cells in three different environments: bulk culture, attachment to a single, flat, impermeable surface, and confinement between two impermeable surfaces (Figure 3A). Cells expressing the α-factor protease, *BAR1* [38], are capable of decreasing the pheromone concentration at their surface [38], [39], so we used *MAT* **a** *bar1Δ* cells for our investigations. We incubated *bar1Δ* cells in 50 nM α-factor in bulk culture for five hours and found that roughly 10% of the cells die (Figure 3B). Although cells grown in bulk culture have no enforced contacts with the other cells or the impermeable surface of the culture tube, it is difficult to control the physical interactions of cells when they are free-floating in liquid culture and possible that cells could stick either to each other, perhaps due to incomplete separation after budding, or to the surface of the culture tube.

To mimic the attachment of two cell walls via mating agglutinins, we attached cells to the impermeable surface of a glass coverslip using the lectin, concanavalin A (ConA), which binds to carbohydrates in the cell wall [40] (Figure 3A). In order to image the yeast cells for an extended period of time, we created a chamber several hundred times the diameter of a yeast cell. Cells were adhered to the ConA-coated coverslip, and the chamber was filled with medium containing 50 nM α-factor using capillary action and then sealed and observed over a period of five hours. We found that *MAT* **a** *bar1Δ* cells attached covalently to an impermeable surface were 1.6 times more likely to die than those in bulk culture, indicating that forced attachment to an impermeable surface increases the rate of cell death (Student's *t*-test, p = 0.01) (Figure 3B).

As cells attached to a single impermeable surface grow, they are free to expand away from the glass coverslip, resulting in a low proportion of the cell wall attached to an impermeable surface and making it likely that cells will polarize away from the impermeable surface. To address this problem we used a second technique to mimic the attachment of two cell walls via mating agglutinins. We trapped cells in a microfluidic chamber whose floor and ceiling are separated by the height of a single yeast cell and through which new medium is constantly perfused (Figure 3A). Cells are loaded into this device and then trapped between the two impermeable surfaces of a silicone ceiling and a glass floor. In addition, as the cells grow, the fraction of their surface that is pressed against the floor and ceiling rises, making them more likely to polarize towards an impermeable surface. Using an inverted microscope, it is possible to image cells over time through the glass floor as medium perfuses through the chamber. Once again we imaged *MAT* **a** *bar1Δ* cells in 50 nM α-factor for five hours (Figure 3C and Movies S1–S3). In the flow chamber, the rate of death of the *MAT* **a** *bar1Δ* cells was more than twice as high as in bulk culture and 1.5 times the rate of death when attached to ConA-coated coverslips, suggesting that a larger area of attachment to an impermeable surface causes increased cell death (Student's *t*-test, p<0.003) (Figure 3B).

### Pheromone-induced cell death increases with increased cell polarization

**Figure 4:**
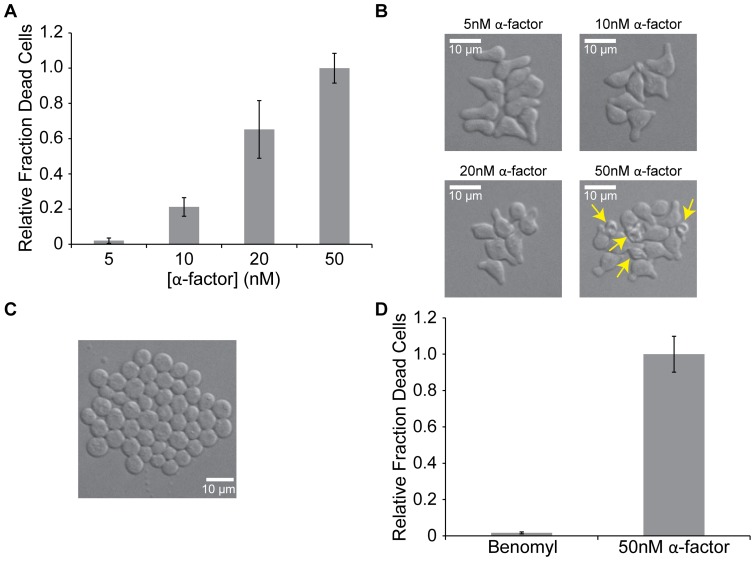
Pheromone-induced cell death increases with increased polarization. **A.** Fraction of *MAT* **a** *bar1Δ* cells that died after five hours exposure to various concentrations of α-factor in the flow chamber relative to the fraction of *MAT* **a** *bar1Δ* cells that died after five hours exposure to 50nM α-factor. Error bars represent the standard deviation of at least three independent experiments. **B.** *MAT* **a** *bar1Δ* cells incubated in the indicated concentration of α-factor for five hours in the flow chamber. Yellow arrows indicate dead cells. The scale bar indicates 10 µm. **C.** *MAT* **a** *bar1Δ* cells exposed to 0.1mM benomyl for five hours in the flow chamber. The scale bar indicates 10 µm. **D.** Fraction of *MAT* **a** *bar1Δ* cells that died after five hours exposure to either 0.1mM benomyl or 50nM α-factor in the flow chamber relative to the fraction of *MAT* **a** *bar1Δ* cells that died after five hours exposure to 50nM α-factor (Student's *t*-test, p<10^−6^). Error bars represent the standard deviation of at least three independent experiments.

Decreased pheromone production has been reported to cause decreased cell fusion [17]. Thus, if the cell death seen here is due to the same activities that normally promote cell fusion, we would expect to see a decrease in cell death with decreased pheromone concentration. We chose to assay the effect of decreased pheromone concentration in the flow chamber, where the highest percentage of cells died when exposed to 50 nM α-factor. As previously reported for cells in bulk culture [37], decreasing the α-factor concentration decreased the percentage of cells that died in the flow chamber. In 5 nM α-factor, 50-fold fewer cells died than when cells were exposed to 50 nM α-factor in the flow chamber (Student's *t*-test, p = 10^−6^), and the fraction of dead cells increased as the concentration of α-factor was increased (Student's *t*-test, p<0.02) (Figure 4A). Although shmoo formation occurs at 5 nM α-factor, as the pheromone concentration was increased, the cells became more tightly polarized, forming pointier shmoos (Figure 4B).

The flow chamber traps cells by wedging them into a space minutely smaller than a single cell in height. When cells are arrested, such as by pheromone stimulation, the cells increase in size as they continue to grow without dividing [41]. Because of this, it is possible that the increased frequency of cell death in the flow chamber, as compared to bulk culture and when cells are attached to ConA-coated coverslips, is not due to the accumulation of enzymes that would normally degrade the cell wall during cell fusion but rather because the physical strain put on the cell wall is too high, which could be increased by the modest pressure (14 kPa = 2 psi) applied to drive the flow of the perfused medium. We therefore used a different method that would arrest the cell cycle without interfering with cell growth. Like pheromone treatment, treating cells with benomyl, a drug that leads to microtubule depolymerization, causes cells to become larger without dividing, but unlike pheromone-arrest, benomyl-arrested cells are unpolarized and arrest in mitosis instead of G1 [42] (Figure 4C). If cells in the flow chamber die because they were squashed, a substantial percentage of benomyl-arrested cells should die in the flow chamber. Although it is possible to find the occasional, dead, benomyl-arrested cell, 60-fold fewer cells die during five hours of benomyl-arrest than during exposure to 50 nM α-factor, indicating that death in the flow chamber is specific to pheromone-arrest, where cells are polarized, and is not due to growth under physical confinement (Figure 4D).

### Pheromone-induced cell death is due to osmotic lysis

Yeast cells require cell walls at least in part due to osmotic pressure. Since the osmolarity of the cytoplasm is higher than the typical extracellular environment, without the rigidity of a cell wall, water would rush into the cell and cause it to lyse [33], and previous studies have shown that cells that are unable to regulate the osmotic balance between the cytoplasm and the extracellular environment have a cell fusion defect [15]. One interpretation of the death of pheromone-treated cells pressed against an impermeable surface is that accumulation of cell wall-degrading enzymes causes the cells to digest part of their cell walls leading to membrane expansion through a hole in the cell wall and eventual lysis. If this interpretation is correct, it should be possible to affect the rate of death by manipulating the osmotic pressure differential between the cell and the medium [43].

**Figure 5:**
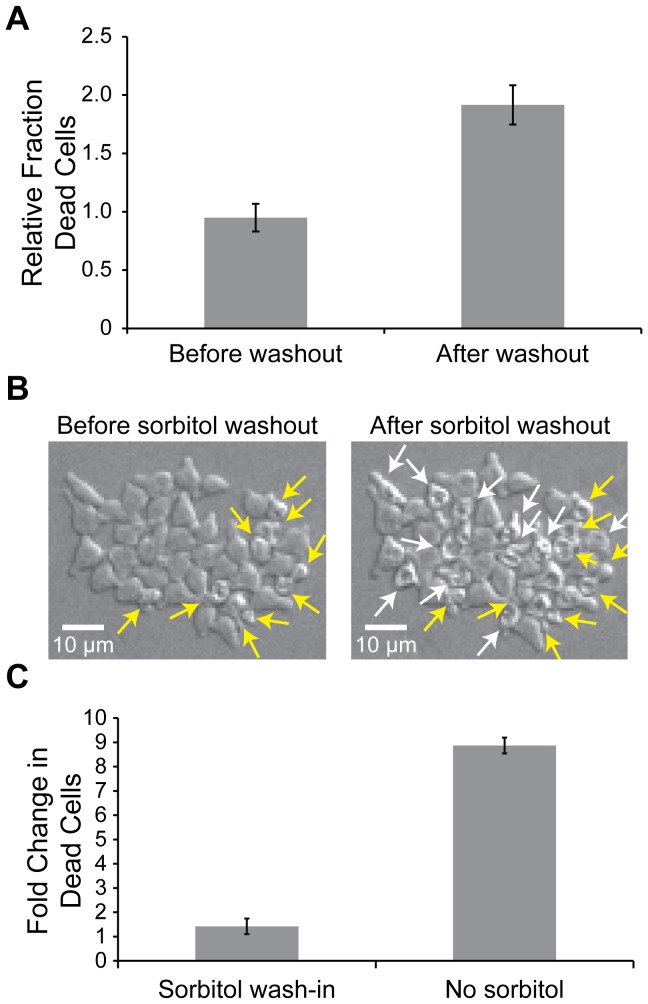
Pheromone-induced cell death is due to osmotic lysis. **A.** *MAT* **a** *bar1Δ* cells were grown in a flow chamber for five hours in medium with 50nM α-factor and 1M sorbitol. After five hours, the sorbitol was washed out, and the cells were incubated in medium with 50nM α-factor and no sorbitol. The fraction of dead cells 10 minutes before and 10 minutes after the 1M sorbitol was washed out relative to the fraction of cells that die when exposed to 50nM α-factor for five hours without the addition of sorbitol was determined (Student's *t*-test, p = 2×10^−4^). Error bars represent the standard deviation of at least three independent experiments. **B.** Cells imaged after 290 minutes in medium with 1M sorbitol and 50nM α-factor (Before sorbitol washout) and 10 minutes after the medium was replaced with medium with 50nM α-factor and no sorbitol (After sorbitol washout). Yellow arrows indicate the cells that died during the 290 minutes of pheromone treatment prior to the sorbitol washout. White arrows in the “After sorbitol washout” picture indicate cells that died during the twenty minute period that spanned the last 10 minutes with sorbitol and the first 10 minutes after the sorbitol washout. The scale bar indicates 10 µm. **C.** Cells were grown in the flow chamber for 80 minutes in medium with 50nM α-factor. After 80 minutes, 1M sorbitol was added to the medium such that the cells were incubated in medium with 1M sorbitol and 50nM α-factor. The fold change in the number of cells that died during the 80 minutes prior to and 60 minutes after the sorbitol wash-in was determined (Sorbitol wash-in). In control chambers (No sorbitol), no sorbitol was added to the medium, and the fold change in the number of cells that died in the two corresponding periods was determined (Student's *t*-test, p = 9×10^−5^). Error bars represent the standard deviation of at least three independent experiments.

We did two experiments to determine whether the pheromone-induced deaths are due to osmotic lysis: either increasing or decreasing the osmotic pressure differential between the cytoplasm and the extracellular environment. We first tested the effect of increasing the osmotic pressure differential between the cytoplasm and the extracellular environment. Cells were exposed to medium with 50 nM α-factor and 1M sorbitol, which increases the osmolarity of the medium, for five hours. When cells are exposed to high external osmolarity, they adapt to the osmotic stress by synthesizing glycerol, which can take place in a matter of minutes [44]–[47], thus increasing their internal osmolarity. Because of this partial restoration of the osmotic pressure gradient across the cell wall, we were not surprised to find that the fraction of cells that die when exposed to 50 nM α-factor and 1M sorbitol is similar to that of cells exposed to only 50 nM α-factor (Figure 5A). Nevertheless, we reasoned that some of the surviving cells would have holes in their cell walls that would be small enough to allow their survival until we increased the osmotic pressure difference between the inside and outside of the cells. Thus, if we replace the sorbitol-containing medium with medium lacking sorbitol, we would expect to see rapid cell death due to the large pressure differential. To test this prediction, we waited until five hours after beginning pheromone treatment and then replaced medium containing 1M sorbitol and 50 nM α-factor with medium containing only 50 nM α-factor. Immediately following the sorbitol washout, the number of dead cells in the flow chamber more than doubled, supporting the idea that the cells in the chamber are dying due to a breach in their cell walls (Figure 5A and 5B and Movie S4).

To test the effect of decreasing the osmotic pressure differential between the cells and the extracellular environment, we exposed cells to 50 nM α-factor in the absence of 1M sorbitol for 80 minutes, at which point cells are just beginning to die (Figure 3C and Movies S1–S3). We determined the percentage of dead cells at this point and then perfused the chamber with medium containing 1M sorbitol and 50 nM α-factor and observed the percentage of dead cells 60 minutes after the change of media. Since the sorbitol is washed in after the cells have begun to shmoo, the cells will have less time to induce the hyperosmotic response, and if the cell death is due to osmotic lysis, we should observe fewer cell deaths when 1M sorbitol is present in the medium. When we observe the fold change in cell death between 80 minutes and 140 minutes after α-factor addition in the absence of 1M sorbitol, there is an 8.9-fold increase in the fraction of dead cells (Figure 5C). However, when 1M sorbitol is added to the medium 80 minutes after α-factor addition, there is only a 1.4-fold increase in the fraction of dead cells between 80 and 140 minutes after α-factor addition, strengthening the evidence that pheromone-induced cell death is due to osmotic lysis (Figure 5C).

### Proteins necessary for cell wall breakdown during mating are required for pheromone-induced cell death

**Figure 6:**
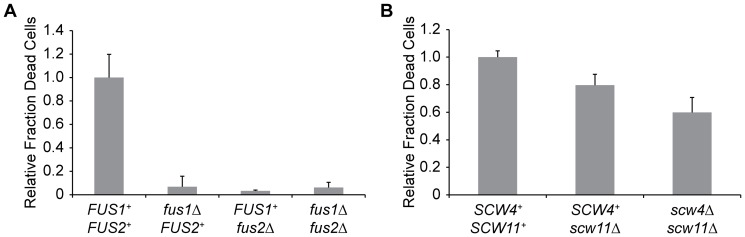
Pheromone-induced cell death is dependent on cell fusion proteins and putative glucanases. **A.** Fraction of dead *MAT* **a** *bar1Δ* cells deleted for different combinations of *FUS1* and *FUS2* relative to the fraction of dead *MAT* **a** *bar1Δ* cells incubated in 50nM α-factor for five hours in the flow chamber. Error bars represent the standard deviation of at least three independent experiments. **B.** Fraction of dead *MAT* **a** *bar1Δ* cells deleted for different combinations of putative cell wall glucanases relative to the fraction of dead *MAT* **a** *bar1Δ* cells incubated in 50nM α-factor for five hours in the flow chamber. Error bars represent the standard deviation of at least three independent experiments.

We investigated the effects of deleting, *FUS1* and *FUS2*, two genes required for efficient cell fusion [24]. When *FUS1*, *FUS2*, or, both *FUS1* and *FUS2* are deleted in both mating partners, prezygotes, consisting of two shmoos bound to each other at their tips, are formed, but cells cannot dissolve their cell walls and thus fail to fuse [24], [28]. Also, in *fus1* and *fus1fus2* mutants, the tightly polarized vesicles that are seen in the fusion zone of wild-type prezygotes and are hypothesized to contain cell wall remodeling enzymes are fewer and more widely dispersed than in wild-type cells [28]. If cell death in the flow chamber is due to pheromone-stimulated cell wall breakdown, mutations known to impair cell wall fusion should reduce the frequency of pheromone-induced cell death events in the flow chamber. Corroborating previous results obtained in bulk cultures [37], deleting *FUS1* and *FUS2* alone and in combination caused more than a 14-fold reduction in cell death in the flow chamber when cells were exposed to 50 nM α-factor for five hours (Student's *t*-test, p<0.002) (Figure 6A).

If the pheromone-induced cell death in the flow chamber is due to holes formed in the cell wall from inappropriate cell wall dissolution, the deletion of cell wall remodeling enzymes should decrease the frequency of pheromone-induced cell death. We investigated the effects of two putative cell wall glucanases that have been implicated in mating: Scw11, a target of pheromone-induced gene expression [19], and its paralog, Scw4, whose deletion interferes with mating [18]. If the observed cell death is due to accumulation of cell-wall degrading enzymes and these glucanases are major contributors to cell wall remodeling during cell wall fusion, deleting them should reduce the frequency of pheromone-induced cell death in the flow chamber. To test this prediction, we incubated *MAT* **a** *bar1*Δ *scw11*Δ cells in a flow chamber in medium containing 50 nM α-factor for five hours. Deleting *SCW11* caused a 20% reduction in cell death compared to *MAT* **a** *bar1*Δ cells (Student's *t*-test, p = 8×10^−4^), and removing both Scw11 and Scw4 caused a 40% reduction in cell death compared to *MAT* **a** *bar1*Δ cells (Student's *t*-test, p = 3×10^−5^) (Figure 6B).

## Discussion

The mating of budding yeast is risky and elaborately choreographed. When two haploid yeast cells mate, they signal through reciprocal pheromones and receptors, stimulate each other to signal ever more strongly, arrest their cell cycles, use pheromone gradients to direct their polarization towards each other, and eventually fuse their cell walls, cell membranes, and nuclei to form a single diploid cell [8], [10], [48], [49]. Although many aspects of yeast mating have been well studied, the mechanism by which cells dissolve their cell walls to allow fusion of their plasma membranes remains mysterious. Cell wall dissolution is a particularly dangerous step in yeast mating. The plasma membranes of the two partner cells cannot touch each other and fuse until the cell walls that lie between them have been dissolved [8]. Because the osmolarity inside a cell is so much higher than outside, the elasticity of the cell wall opposes the osmotic pressure difference between the cytoplasm and the environment, thus keeping water from rushing into the cell and causing it to lyse. A cell that dissolves any part of its cell wall that does not touch a closely apposed mating partner will die [15], [16].

Pheromone-induced cell death was studied previously [36], [37] and hypothesized to be due to inappropriate activation of cell fusion machinery, resulting in cell wall dissolution and eventual cell lysis [37]. Although it was observed that this lysis can be reduced by increasing cell wall integrity and deleting certain proteins involved in cell fusion, a hypothesis to explain why cells were dissolving their cell walls was not given [37]. Many hypotheses can be generated to explain how cell wall dissolution is regulated in time and space to promote mating and prevent accidental deaths. Most of them posit additional signaling systems in addition to the known mechanisms of pheromone signaling, but no additional signaling molecules have been uncovered, despite a variety of searches [8], [28], [50], [51]. The failure of these attempts led us to propose a hypothesis that requires no new components and instead appeals to the physical differences between mating cell pairs and isolated, pheromone-stimulated cells.

In an isotropically growing cell, cell wall synthesizing and remodeling enzymes are secreted uniformly around the cell, whereas the polarized growth that accompanies both budding and shmooing requires similarly polarized secretion of these enzymes [33] (Figure 1A and 1B). Thus we hypothesize that cell wall remodeling enzymes, such as Scw4 and Scw11, are preferentially released at the shmoo tip, which locally weakens the cell wall, allowing the shmoo to grow continuously up the pheromone gradient. As a shmoo approaches a suitable partner, the concentration of pheromone increases, tightening the polarization, and increasing the concentration of cell wall remodeling enzymes in the part of the cell wall that has polarized towards its partner's site of maximum pheromone secretion [49], [52]–[55]. If the remodeling enzymes are diffusible, the maximum concentration they can reach in a shmoo that has not bound to a partner is limited: even though the secretion rate of cell wall remodeling enzymes is high, the enzymes are able to diffuse through the cell wall, keeping their concentration in the range that is high enough to allow rapid remodeling of the growing shmoo but low enough to prevent cell wall rupture (Figure 1C). But when two shmoo tips are attached to each other via mating agglutinins, it takes longer for cell wall remodeling enzymes to diffuse out of the fusion zone because they must now travel laterally through the cell wall in order to escape, thus increasing the local concentration of the remodeling enzymes and leading to the gradual dissolution of the cell wall, exposing the two plasma membranes to each other and allowing their fusion to create a single, diploid cell (Figure 1D).

Our data supports the hypothesis that increased secretion of remodeling enzymes and the longer distances they have to diffuse when two polarization zones agglutinate to each other causes cell wall dissolution. We tightly apposed pheromone-treated cells to an impermeable surface, mimicking the effect of cell-cell attachment during mating while ensuring that the only signal these cells can receive is a uniformly high pheromone concentration (Figure 1E). The cell lysis events we observe are not due merely to the physical constraints of a flow chamber: cells also lyse when they are chemically attached to a glass coverslip with essentially infinite space to expand, and confined cells that grow larger isotropically while they are arrested in mitosis do not lyse [42]. By manipulating the presence of an osmoprotectant in the media, we show that the frequency of lysis events rises with increasing osmotic pressure difference between the osmolarity of the cell and its environment, implying that breaches in the cell wall lead to osmotically induced lysis. Lysis depends on Fus1 and Fus2, proteins that have been previously implicated in cell fusion [24], and the cell wall dissolution appears to be at least partially accomplished by two putative glucanases, Scw4 and Scw11.

Although a contact-driven increase in the concentration of cell wall remodeling enzymes is the simplest explanation for cell wall dissolution during mating, other viable hypotheses exist. One is that cells can only dissolve their cell walls in response to a high concentration of pheromone that they experience while attached to a mating partner. When cells are in the flow chamber, we do see an increase in cell lysis as we increase the pheromone concentration arguing that high pheromone concentrations promote cell wall dissolution; however, the increase in cell lysis in cells apposed to impermeable surfaces argues that factors other than pheromone concentration contribute to cell wall dissolution. Another hypothesis is that the cell is capable of detecting cell wall or cell membrane deformations that indicate that two shmoo tips are attached via mating agglutinins. Our experiments do not negate this possibility, since it is likely that contact with impermeable surfaces causes cell wall deformation, but the failure of previous attempts to identify additional signaling components [8], [28], [50], [51] argues against this model. A third possibility is that cells respond to a direct signal from another cell, such as an additional, uncharacterized signaling mechanism similar to the G-protein coupled receptors involved in pheromone stimulation, or, perhaps, the oscillation in pheromone concentration that would occur if cells were close enough to detect the pulses of increased pheromone concentration concomitant with the fusing of individual secretory vesicles with the plasma membrane. The fact that lysis occurs without the presence of a mating partner argues against any hypothesis that requires communication aside from that of the reciprocal pheromones and pheromone receptors between the two mating cells.

Taken together with previously published studies, our data supports a model that involves pheromone-induced, polarized secretion of cell wall remodeling enzymes. When cells are pheromone stimulated, a MAP kinase cascade activates transcription of pheromone-induced genes [10]. Along with many others, these genes include the expression of mating agglutinins and cell wall remodeling enzymes, which are packaged into vesicles for secretion into the extracellular environment [11]–[13], [17]–[19]. Fus2 and Rvs161, a protein that binds to curved membranes [25], [26] and is involved in cell fusion [51], bind to these vesicles and travel along actin cables to the site of polarization in a Myo2-dependent fashion [56] where they are anchored to the plasma membrane by Fus1 [23], which interacts with the polarisome [30]. Fus2 and Rvs161 in conjunction with Cdc42 may then facilitate the fusion of these vesicles with the plasma membrane [57].

When cells are weakly stimulated, they form broad shmoos (Figure 4B). Although these cells are polarized, the zone of polarization is relatively large, and presumably, the vesicles containing cell wall remodeling enzymes are released into a relatively large area. The enzymes cleave carbohydrate bonds as they diffuse through the cell wall matrix, weakening the cell wall and allowing for further expansion in the direction of highest pheromone concentration [34]. As a shmoo gets closer to a cell of the opposite mating type, the pheromone concentration increases and the shmoo tip becomes more tightly polarized [53], [54]. This tighter polarization focuses the secretion of cell wall remodeling enzymes into a smaller fraction of the cell surface, increasing the concentration of cell wall remodeling enzymes in this zone. Although the concentration of cell wall remodeling enzymes in this zone has increased, it is not typically high enough to cause dissolution of the cell wall unless the shmoo tip is pressed against an impermeable barrier, forcing the enzymes to travel further to reach bulk solution. Since the time taken to diffuse a given distance rises with the square of the distance, the effective speed at which the enzymes move falls, and thus their concentration in the cell wall rises (Figure 1 and 2). Similarly, when the two polarized cells attach at their shmoo tips via mating agglutinins, the presence of a second cell membrane traps the remodeling enzymes in the cell wall by requiring them to move laterally along the cell surface to exit the cell wall, increasing their concentration and breaking down the cell wall (Figure 1D). As the wall dissolves, the two plasma membranes come into contact with one another, allowing membrane fusion to begin and pushing the Fus2-bound vesicles outward [23], which allows for the rest of the intervening cell wall to be dissolved and eventually full fusion of the newly formed zygote.

Understanding more about the cell fusion of budding yeast is an important step in understanding cell fusion in more complex organisms. Although animal cells do not have a cell wall, the extracellular matrix surrounding these cells must be dissolved prior to cell fusion. A similar local increase in enzyme concentration at points where cells are very close to each other could promote the digestion of the matrix and allow the plasma membranes of the two partners to touch each other.

## Materials and Methods

### Yeast strains and culturing

Strains used in this study are listed in Table 1. All strains were derived from the W303 background [58] (*ade2-1 can1-100 his3-11,15 leu2-112 trp1-1 ura3-1*) using standard genetic techniques. All media was prepared as described [59] and contained 2% wt/vol of glucose. Cells were either grown in Synthetic Complete medium (SC) or Yeast Extract Peptone Dextrose (YPD) at 30°C in culture tubes on roller drums or at room temperature (25°C) for timelapse microscopy. Bovine serum albumin (BSA) was used to reduce the non-specific absorption of α-factor to glass and plastic surfaces; it was made into 10% wt/vol stocks in deionized water and then diluted into media to 0.1% wt/vol. Synthetic α-factor (Biosynthesis, Lewisville, TX) was suspended in dimethyl sulfoxide (DMSO) and then diluted into YPD+0.1% BSA or SC+0.1% BSA at the appropriate concentration. When appropriate, 1M sorbitol was added to YPD by dissolving sorbitol into YPD. YPD containing 1-(butylcarbomoyl)-2-benzimidasolecarbamate (benomyl) was prepared by heating YPD to 65°C and adding 34 mM benomyl in DMSO dropwise to a final concentration of 0.1 mM. Yeast extract was obtained from EMD Millipore (Billerica, MA). Peptone and yeast nitrogen base were obtained from BD (Franklin Lakes, NJ). Bacto-agar was obtained from US Biological (Swampscott, MA). Unless otherwise noted, all chemicals were obtained from Sigma-Aldrich (St. Louis, MO).

**Table 1:** Strains used in this study.

Strain Name	Genotype (all cells are in the W303 background)
LBHY52	*MAT*a *bar1Δ::KanMX6 P_ACT1_-yCerulean-HIS3MX6 @ P_ACT1_*
LBHY77	*MAT*a *bar1Δ::KanMX6 fus1Δ::NatMX4 P_ACT1_-yCerulean-HISMX3 @ P_ACT1_*
LBHY80	*MAT*a *bar1Δ::KanMX6 fus2Δ::HphMX4 P_ACT1_-yCerulean-HISMX3 @ P_ACT1_*
LBHY84	*MAT*a *bar1Δ::KanMX6 fus1Δ::NatMX4 fus2Δ::HphMX4 P_ACT1_-yCerulean-HISMX3 @ P_ACT1_*
LBHY136	*MAT*a *bar1Δ::ADE2 SPA2-YFP:HIS3 scw11Δ::HphMX4 ade2-1 can1-100 his3-11,15 leu2-3,112 trp1-1 ura3-1*
LBHY153	*MAT*a *bar1Δ::ADE2 SPA2-YFP:HIS3 scw4Δ::KanMX6 scw11Δ::HphMX4 ade2-1 can1-100 his3-11,15 leu2-3,112 trp1-1 ura3-1*
MP 384	*MAT*a *bar1Δ::ADE2 SPA2-YFP:HIS3 ade2-1 can1-100 his3-11,15 leu2-3,112 trp1-1 ura3-1*

All strains are from this study except for MP 384, which is from M. Piel.

### Microscopy

Microscopy was done at room temperature using a Nikon Ti-E inverted microscope with a 20x Plan Apo VC 0.75NA air lens, and images were acquired with a Photometrics CoolSNAP HQ camera (Roper Scientific, AZ). Timelapse photography was done using Metamorph 7.7 (Molecular Devices, CA); pictures were acquired using differential interference contrast every 10 minutes with a 10 ms exposure.

### Bulk culture lysis assay

Cells were grown to log phase (∼5×10^6^ cells/mL) at 30°C in YPD and counted using a Z2 Coulter counter (Beckman-Coulter, CA). Cells were washed in YPD+0.1% BSA and resuspended at 10^6^ cells/mL into plastic 14 mL culture tubes (BD Falcon, MA) in YPD+0.1% BSA with 50 nM α-factor. These cultures were then incubated on a roller drum at 30°C for five hours. Cells were then put directly onto glass slides (Corning, NY) with uncoated coverslips (VWR, PA) and imaged at 20x magnification using differential interference contrast with a 10 ms exposure. Prior to the experiment, the plastic culture tubes were coated in BSA by incubating overnight at 4°C with phosphate buffered saline (PBS) with 2% wt/vol BSA. The PBS+2% BSA was poured out immediately prior to the addition of the cell cultures. To determine the percentage of cells that lysed, more than 50 cells were counted from each trial. Statistical significance was determined using Student's *t*-Test.

### Concanavalin-A coated coverslip lysis assay

Coverslips (VWR, PA) were coated in concanavalin A (MP Biomedicals, OH) in a protocol modified from Joglekar *et al.* (2008) [60]. Briefly, coverslips were soaked in 1M NaOH for one hour at room temperature (25°C), rinsed five times with deionized, filtered water, and then incubated at room temperature for one hour in a solution of 10 mM Na_2_HPO_4_ pH 6.0 (Fisher Biotech, MA) +1 mM CaCl_2_+0.5 mg/mL concanavalin A. Coverslips were then rinsed five times with deionized, filtered water and air-dried over a 100°C heat block. To make a chamber, strips of parafilm (American National Can, IL) were melted at 100°C on glass slides (Corning, NY) and concanavalin A-coated coverslips were placed on top of the strips. The parafilm was allowed to cool to room temperature, creating channels with a glass slide ceiling, concanavalin A-coated coverslip floor, and parafilm walls on two, parallel sides.

Cells were grown to log phase (∼5×10^6^ cells/mL) at 30°C in YPD and then washed in SC+0.1% BSA. 50 nM α-factor was added to the cells, and the cells were immediately injected into the chamber using capillary action. The cells were allowed to adhere to the concanavalin A-coated coverslip for 10 minutes, and then 200 µL of SC+0.1% BSA with 50 nM α-factor was flowed through the chamber using capillary action to wash off excess cells. The chamber was sealed with candle wax and imaged at 20x magnification, using differential interference contrast with a 10 ms exposure, every 10 minutes for five hours from the time when the cells were exposed to α-factor-containing medium. To determine the percentage of cells that lysed, more than 400 cells were counted from each trial. Statistical significance was determined using Student's *t*-Test.

### Flow chamber lysis assay

Cells were grown to log phase (∼5×10^6^ cells/mL) at 30°C in YPD and then washed in YPD+0.1% BSA. For experiments involving α-factor, the microfluidic chambers (CellAsic, Hayward, CA) [61] were pretreated by perfusing PBS+2% BSA through the chamber at 34 kPa (5 psi) for 10 minutes and then YPD+0.1% BSA through the chamber at 34 kPa (5 psi) for 10 minutes. After cells were loaded, YPD+0.1% BSA with the appropriate concentration of α-factor was perfused through the chamber at 14 kPa (2 psi), and pictures were taken at 20x magnification every 10 minutes for five hours using differential interference contrast with a 10 ms exposure.

For experiments involving benomyl, the microfluidic chambers were pretreated by perfusing YPD through the chamber at 34 kPa (5 psi) for 10 minutes. After the cells were loaded, YPD+0.1 mM benomyl was perfused through the chamber at 14 kPa (2 psi), and pictures were taken at 20x magnification every 10 minutes for five hours using differential interference contrast with a 10 ms exposure. To determine the percentage of cells that lysed, more than 250 cells were counted from each trial. Statistical significance was determined using Student's *t*-Test.

### Sorbitol wash-out assay

Cells were grown to log phase (∼5×10^6^ cells/mL) at 30°C in YPD and then washed in YPD+0.1% BSA. The microfluidic chambers (CellAsic, Hayward, CA) [61] were pretreated by perfusing PBS+2% BSA through the chamber at 34 kPa (5 psi) for 10 minutes and then YPD+1M sorbitol +0.1% BSA through the chamber at 34 kPa (5 psi) for 10 minutes. After cells were loaded, YPD+1M sorbitol +0.1% BSA+50 nM α-factor was perfused through the chamber at 14 kPa (2 psi) for five hours. After five hours, the medium containing 1M sorbitol was washed out, and YPD+0.1% BSA+50 nM α-factor was perfused through the chamber at 14 kPa (2 psi) for two hours. Pictures were taken at 20x magnification every 10 minutes for seven hours using differential interference contrast with a 10 ms exposure. To determine the percentage of cells that lysed, more than 500 cells were counted from each trial. Statistical significance was determined using Student's *t*-Test.

### Sorbitol wash-in assay

Cells were grown to log phase (∼5×10^6^ cells/mL) at 30°C in YPD and then washed in YPD+0.1% BSA. The microfluidic chambers (CellAsic, Hayward, CA) [61] were pretreated by perfusing PBS+2% BSA through the chamber at 34 kPa (5 psi) for 10 minutes and then YPD+0.1% BSA at 34 kPa (5 psi) through the chamber for 10 minutes. After cells were loaded, YPD+0.1% BSA+50 nM α-factor was perfused through the chamber at 14 kPa (2 psi) for 80 minutes. At the end of 80 minutes, the YPD+0.1% BSA+50 nM α-factor was washed out, and YPD+1M sorbitol +0.1% BSA+50 nM α-factor was perfused through the chamber at 14 kPa (2 psi) for 60 minutes. Pictures were taken at 20x magnification every 10 minutes for 140 minutes using differential interference contrast with a 10 ms exposure. To determine the percentage of cells that lysed, more than 600 cells were counted from each trial. Statistical significance was determined using Student's *t*-Test.

## Supporting Information

Movie S1 **Pheromone-induced cell death in the flow chamber.** *MAT* **a** *bar1Δ* cells were incubated in medium containing 50 nM α-factor for five hours in the flow chamber. White arrows indicate cells that die during the movie. Cells were imaged every 10 minutes. The scale bar indicates 10 µm. Each movie is from an independent experiment.
(MP4)

Movie S2 **Pheromone-induced cell death in the flow chamber.** *MAT* **a** *bar1Δ* cells were incubated in medium containing 50 nM α-factor for five hours in the flow chamber. White arrows indicate cells that die during the movie. Cells were imaged every 10 minutes. The scale bar indicates 10 µm. Each movie is from an independent experiment.
(MP4)

Movie S3 **Pheromone-induced cell death in the flow chamber.** *MAT* **a** *bar1Δ* cells were incubated in medium containing 50nM α-factor for five hours in the flow chamber. White arrows indicate cells that die during the movie. Cells were imaged every 10 minutes. The scale bar indicates 10 µm. Each movie is from an independent experiment.
(MP4)

Movie S4 **Pheromone-induced cell death is due to osmotic lysis.** *MAT* **a** *bar1Δ* cells were incubated in the flow chamber for five hours in medium containing 50 nM α-factor and 1M sorbitol. After five hours, the sorbitol was washed out, and the cells were incubated in medium containing 50 nM α-factor and no sorbitol. Cells were imaged every 10 minutes. The scale bar indicates 10 µm.
(MP4)
